# Inflammation induced by the new lineage of *Vibrio cholerae* serogroup O1 in the neonatal mouse model

**DOI:** 10.3389/fimmu.2025.1617803

**Published:** 2025-10-03

**Authors:** Xiuping Fu, Jingyun Zhang, Weili Liang, Baowei Diao, Bo Pang, Biao Kan

**Affiliations:** State Key Laboratory of Infectious Disease Prevention and Control. National Institute for Communicable Disease Control and Prevention, Chinese Center for Disease Control and Prevention, Beijing, China

**Keywords:** *Vibrio cholerae*, inflammation, cytokines and chemokines, colonization, the neonatal mouse

## Abstract

**Background:**

A novel lineage of serogroup O1 El Tor *V. cholerae*, genetically distinct from the seventh pandemic strain, has recently been identified in China and linked to diarrheal outbreaks. Investigations were conducted to examine the inflammation in the intestines of mice infected with *V. cholerae* strains including the new lineage strains VC6050 (*ctxAB* +) and VC6055 (*ctxAB* -), as well as the seventh pandemic strains of *V. cholerae* N16961.

**Results:**

The result showed that the colonization abilities of *V. cholerae* in the intestines of mice infected with VC6050 and N16961 was significantly higher than that of the VC6055 group. Histological sections of the small intestine revealed a few inflammatory cell infiltrations in the muscularismucosa, with inflammation being the primary form of tissue damage. The transcript changes in the neonatal mouse intestine were primarily associated with immune and inflammation-related genes after *V. cholerae* infection, including CCL7, CCL17, CCL21, CXCL9, and CXCL10. In comparison to the seventh pandemic strain N16961, the new lineage strains exhibited significant up-regulation of carboxyesterase and genes involved in aquaporin-mediated transport, whereas some inflammation-related genes were down-regulated. When compared to the nontoxigenic strain VC6055, the toxigenic strains N16961 and VC6050 demonstrated significant up-regulation of inflammation related genes and alpha-defensin gene (Defa). Conclusions: The results suggest that, in comparison to the seventh pandemic strains of *V. cholerae*, the new lineage strains exhibit lower levels of inflammatory cytokines and chemokines. Furthermore, CTX-positive strains, when contrasted with CTX-negative strains, not only activate a greater number of inflammatory factors but also stimulate the host to generate more antimicrobial peptides.

## Introduction

*Vibrio cholerae* is an important human intestinal pathogen that persists in aquatic reservoirs and plankton. Based on their O-antigen structure, over 200 serogroups of *V. cholerae* have been identified; however, only serogroups O1 and O139, which carry the cholera toxin-encoding phage (CTXΦ), are associated with pandemic cholera (1–3). The serogroup O1 *V. cholerae* is further divided into two biotypes: the classical biotype and the El Tor biotype. Within the serogroup O1 El Tor *V. cholerae* population, several lineages have been identified, one of which was responsible for the seventh global cholera pandemic and is characterized by a highly clonal genome structure (4–6). In recent years, enhanced surveillance efforts have led to the discovery of a novel lineage of serogroup O1 El Tor *V. cholerae* in China. This newly identified lineage has been associated with diarrhea cases in several provinces and municipalities surrounding the Bohai Bay, including Beijing. This lineage *V. cholerae* belongs to a distinct genetic branch, separate from the strains responsible for the ongoing seventh cholera pandemic. It exhibits unique genomic characteristics that differentiate it from the pandemic strains.

Cholera toxin (CT), encoded by the *ctxAB* genes, causes the typical symptom of cholera—severe secretory diarrhea—and is the primary virulence factor of *V. cholerae* (7). Nontoxigenic strains of *V. cholerae* that lack the *ctxAB* genes are incapable of producing the classical CT, but they can still cause diseases, including sporadic watery diarrhea and inflammatory enterocolitis, and sometimes lead to outbreaks (8, 9). While most strains in the new lineage lack the *ctxAB* genes, the presence of *ctxAB* genes in some strains indicates the potential of this new lineage to cause cholera outbreaks (10).

Although cholera is commonly considered a non-inflammatory secretory disease, innate immune responses have been observed in cholera patients during the acute phase of infection (11, 12). Additionally, CT induces strong mucosal and systemic antibody responses, resulting in long-lasting protection against cholera (12–14). The inflammatory response induced by *V. cholerae* has been investigated using both adult mouse pulmonary infection models and neonatal mouse models (14, 15). Colonizing adult mice orally with *V. cholerae* is challenging due to their developed immune systems; however, chronic colonization can be sustained through antibiotic treatment (16). In contrast, neonatal mice, which lack a fully functional adaptive immune system, can be readily colonized by *V. cholerae* following oral inoculation, leading to an acute intestinal infection that mimics severe cholera in humans (15). This feature of neonatal mice allows for the isolated study of the innate immune response. In this study, adult Balb/c mice and C57BL/6 suckling mice were used to examine the characteristics of the innate immune response in the intestines of mice infected with vari*ous strains of V. chol*erae, including the new lineage strains VC6050 (*ctxAB* ^+)^ and VC6055 (*ctxAB* ^-^), as well as the representative seventh pandemic strain N16961 (*ctx*AB +).Our findings will provide essential experimental evidence to enhance our understanding of the infection mechanisms and pathophysiology of the new lineage of *V. cholerae*.

## Materials and methods

### Mice

C57BL/6 neonatal mice aged 5–6 days and female BALB/c mice aged 6–8 weeks were housed in an Animal Biosafety Level 2 (ABSL-2) laboratory under Specific Pathogen Free (SPF) conditions. The mice were maintained on a 12-hour light/12-hour dark cycle and had ad libitum access to food and water throughout the experiment.

### Bacterial strains

The following strains were used: the seventh pandemic strain N16961 (O1 El Tor biotype, *ctxAB* ^+^), and two strains from the new lineage, VC6050 (O1 Group, *ctxAB* ^+^) and VC6055 (O1 Group, *ctxAB* −).

### *V. cholerae* infection of infant mice

Eight litters of C57BL/6 neonatal mice (7–8 pups per litter), aged 5–6 days, were randomly assigned to four experimental groups. Three groups were orally inoculated with suspensions of different *V. cholerae* strains (N16961, VC6050, and VC6055), while one group received phosphate-buffered saline (PBS) as a control. The *V. cholerae* strains were cultured in Luria-Bertani (LB) broth at 37°C. Bacterial cultures were harvested by centrifugation, washed, and resuspended in PBS to an optical density at 600 nm (OD600) of 1.0. Each mouse was administered 100 μL of the bacterial suspension, containing approximately 10^8^ cells, via intragastric gavage. After 24 hours, the mice were euthanized, and their small intestines were collected in 5 mL of PBS. The small intestines from five mice in each group were homogenized. Serial dilutions of the homogenates were then plated on thiosulfate-citrate-bile-salts (TCBS) agar plates to enumerate *V. cholerae* cells, thereby assessing the colonization ability of each strain. The small intestines from another five mice in each group were subjected to histopathological analysis. The remaining five small intestines from each group were reserved for RNA sequencing.

### Cytokine assay

Female BALB/c mice (6–8 weeks old) were orally inoculated with the *V. cholerae* strain N16961, with PBS used as a control. At 4 hours and 24 hours post-inoculation, groups of mice were euthanized, and blood was collected from the heart to obtain serum samples. Cytokine levels were measured using the BD MS TH1-TH2-TH17 Cytokine Bead Array (CBA) Kit (detecting IL-2, IL-4, IL-6, IL-10, IL-17A, TNF, and IFN-γ) on a BD FACSAria III flow cytometer. A standard curve was generated using standard concentrations ranging from 0.00 pg/mL to 5,000.00 pg/mL, with the following points: 0.00, 19.53, 39.06, 78.13, 156.25, 312.50, 625.00, 1,250.00, 2,500.00, and 5,000.00 pg/mL.

### Histological analysis by HE staining

Small intestinal tissues from 5–6-day-old C57BL/6 neonatal mice were collected and immediately placed in 4% paraformaldehyde solution for fixation at 4°C for 24 hours. Following fixation, the samples were embedded in paraffin. Tissue sections were prepared in a longitudinal orientation and stained with hematoxylin and eosin (HE) for histological examination and panoramic scanning. The histopathological score was calculated as follows: (Inflammatory Response + Lesion Depth + Crypt Destruction) × Lesion Extent. Inflammatory Response: 0 points - None, 1 point - Mild, 2 points - Moderate, 3 points - Severe. Lesion Invasion Depth: 0 points - None, 1 point - Submucosa, 2 points - Muscular layer, 3 points - Serosal layer. Crypt Destruction: 0 points - None, 1 point - Basal one-third of crypts destroyed, 2 points - Basal two-thirds of crypts destroyed, 3 points - Entire crypts destroyed with intact epithelium, 4 points - Entire crypts and epithelium destroyed. Lesion Extent: 0 points - <1%, 1 point - 1%–25%, 2 points - 26%–50%; 3 points - 51%–75%, 4 points - 76%–100%.

### RNA extraction

Total RNA was extracted from small intestinal tissues using TRIzol^®^ Reagent according to the manufacturer’s instructions. RNA quality was assessed using a 5300 Bioanalyzer (Agilent), and RNA concentration was quantified using the ND-2000 spectrophotometer (NanoDrop Technologies). Only high-quality RNA samples (OD260/280: 1.8-2.2; OD260/230: ≥2.0; RQN: ≥6.5; 28S:18S: ≥1.0; quantity: >1 μg) were selected for constructing the sequencing library.

### RNA sequencing

The RNA-seq transcriptome library was prepared using the Illumina^®^ Stranded mRNA Prep, Ligation Kit (San Diego, CA) with 1 μg of total RNA. Briefly, messenger RNA (mRNA) was isolated using the poly(A) selection method with oligo(dT) beads and then fragmented using fragmentation buffer. Double-stranded cDNA was synthesized using the SuperScript™ Double-Stranded cDNA Synthesis Kit (Invitrogen, CA) with random hexamer primers. The synthesized cDNA was subjected to end-repair, phosphorylation, and adapter ligation according to the library construction protocol. Libraries were size-selected for cDNA target fragments of 300 bp on 2% Low Range Ultra Agarose (Bio-Rad), followed by PCR amplification using Phusion DNA Polymerase (NEB) for 15 cycles. After quantification using Qubit 4.0, the sequencing library was sequenced on the NovaSeq X Plus platform (PE150) using the NovaSeq Reagent Kit.

### Differential expression analysis, functional enrichment and protein-protein interaction analysis

To identify differentially expressed genes (DEGs) between two samples, the expression level of each transcript was calculated using the transcripts per million reads (TPM) method. Gene abundances were quantified using RSEM. Differential expression analysis was performed using the DESeq2 (17) packages in R. DEGs with an absolute log2 fold change (|log2FC|) ≥ 1 and a false discovery rate (FDR) ≤ 0.05 (DESeq2) or FDR ≤ 0.001 (DEGseq) were considered to be significantly differentially expressed.

Functional enrichment analysis was performed to identify significantly enriched Gene Ontology (GO) terms and Kyoto Encyclopedia of Genes and Genomes (KEGG) pathways among the DEGs. The analysis employed the Bonferroni correction, with a significance threshold of P-value ≤ 0.05 compared to the whole-transcriptome background. The GO functional enrichment analysis was conducted using the Goatools package, while the KEGG pathway analysis was performed using the Python SciPy library.

Furthermore, a PPI network was constructed to investigate the gene-gene interaction patterns among the identified DEGs. The PPI network was built using STRING database interactions and visualized with Cytoscape software. Network analysis was performed to identify key modules and hub genes involved in the biological processes of interest.

### Reverse transcription quantitative real-time PCR

To validate the transcriptome data, we selected six DEGs for RT-qPCR analysis. Total RNA was extracted from small intestinal tissues using the RNeasy Mini Kit (Qiagen). Genomic DNA was removed by incubation with DNase I (Promega). First-strand cDNA was synthesized from 1 μg of RNA using the Superscript III First-Strand Synthesis System (Invitrogen) with oligo(dT) primers. qPCR analysis was performed using the SYBR Green EX Taq Mix (TaKaRa) on a Bio-Rad CFX96 Real-Time PCR Detection System. The relative expression levels of target genes were determined using the 2^−ΔΔCq^ method, with GAPDH as the internal control. Each qPCR reaction was performed in triplicate, and the experiment was repeated twice to ensure reproducibility. Primer sequences are listed in **Supplementary Table S1**.

## Results

### Differential colonization but similar histopathological impact of three *V. cholerae* strains in neonatal mouse intestines

**Figure 1:**
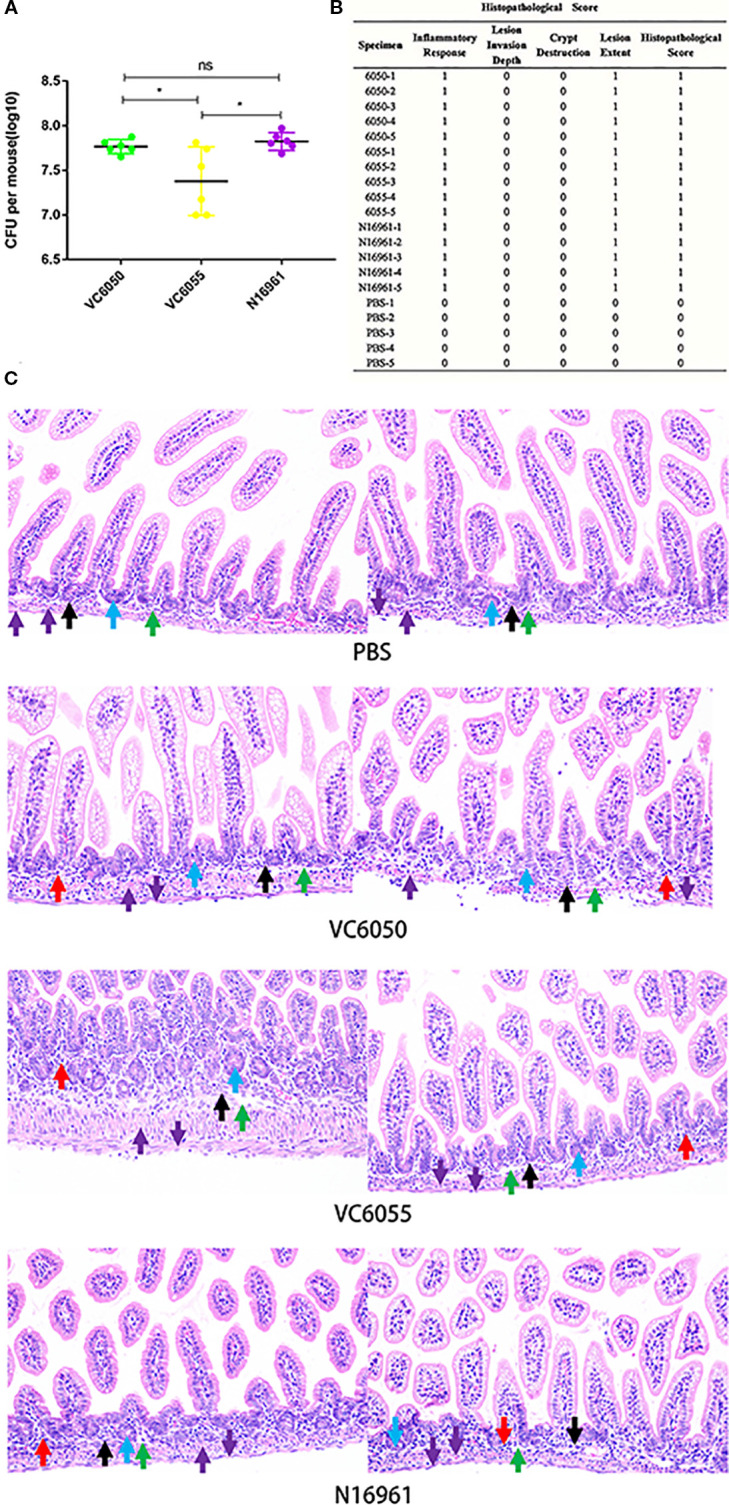
**(A)** Colonization of *V. cholerae* strains VC6050, VC6055, and N16961 in the small intestine of C57BL/6 neonatal mice. *P<0.01; unpaired two-tailed t-test. **(B)** Histopathological Score of *V. cholerae* strains VC6050, VC6055, and N16961. **(C)** Histological sections of the small intestine of C57BL/6 neonatal mice 24 hours post- infection, magnified at 20x. Control group (PBS): each mouse was gavaged with 100 μL of PBS. Experimental group (VC6050, VC6055, N16961): each mouse was gavaged with 10^8^ CFU of *V. choleare*. Blue arrows indicate crypts; red arrows, inflammatory cells; purple arrows, muscularis mucosae; green arrows, submucosa; black arrows, muscularis propria.

C57BL/6 neonatal mice were orally inoculated with *V. cholerae* strains VC6050, VC6055, and N16961, with PBS as a control. At 24 hours post-inoculation, intestinal colonization levels were significantly higher in mice infected with VC6050 and N16961 than in those infected with VC6055. No significant difference was observed between the VC6050 and N16961 groups (**Figure 1A**).

Histopathological examination revealed mild structural abnormalities in the intestinal tissues of mice infected with the three strains. These abnormalities included a few inflammatory cell infiltrations in the muscularis mucosa, as indicated by the red arrows (**Figure 1C**). Inflammation was the primary form of tissue damage observed. In contrast, the intestinal tissue structure in the PBS control group remained largely normal. No significant pathological differences were observed among the histopathological score of VC6050, VC6055, and N16961 groups (**Figure 1B**).

### RNA-seq

In the present study, DEG analysis was conducted to elucidate the transcriptional changes in the neonatal mouse intestine following infection with various strains of *V. cholerae*. A total of 20 transcriptome samples were sequenced, generating 171.97 Gb of clean data. Each sample yielded at least 5.89 Gb of clean data, with a Q30 base percentage exceeding 96.38%. Compared to the PBS control group, the number of DEGS for strains N16961, VC6050, and VC6055 were 1274, 796 and 220 respectively. In pairwise comparisons among N16961, VC6050, and VC6055, the numbers of DEGS were 1134, 1111 and 726 respectively (**Table 1**).

**Table 1:** Summary of differentially expressed genes.

Diff_group	Total DEG	Up	Down
N16961_vs_PBS	1274	744	530
N16961_vs_VC6050	1134	346	788
N16961_vs_VC6055	1111	649	462
VC6050_vs_PBS	796	614	182
VC6050_vs_VC6055	726	545	181
VC6055_vs_PBS	220	119	101

**Figure 2:**
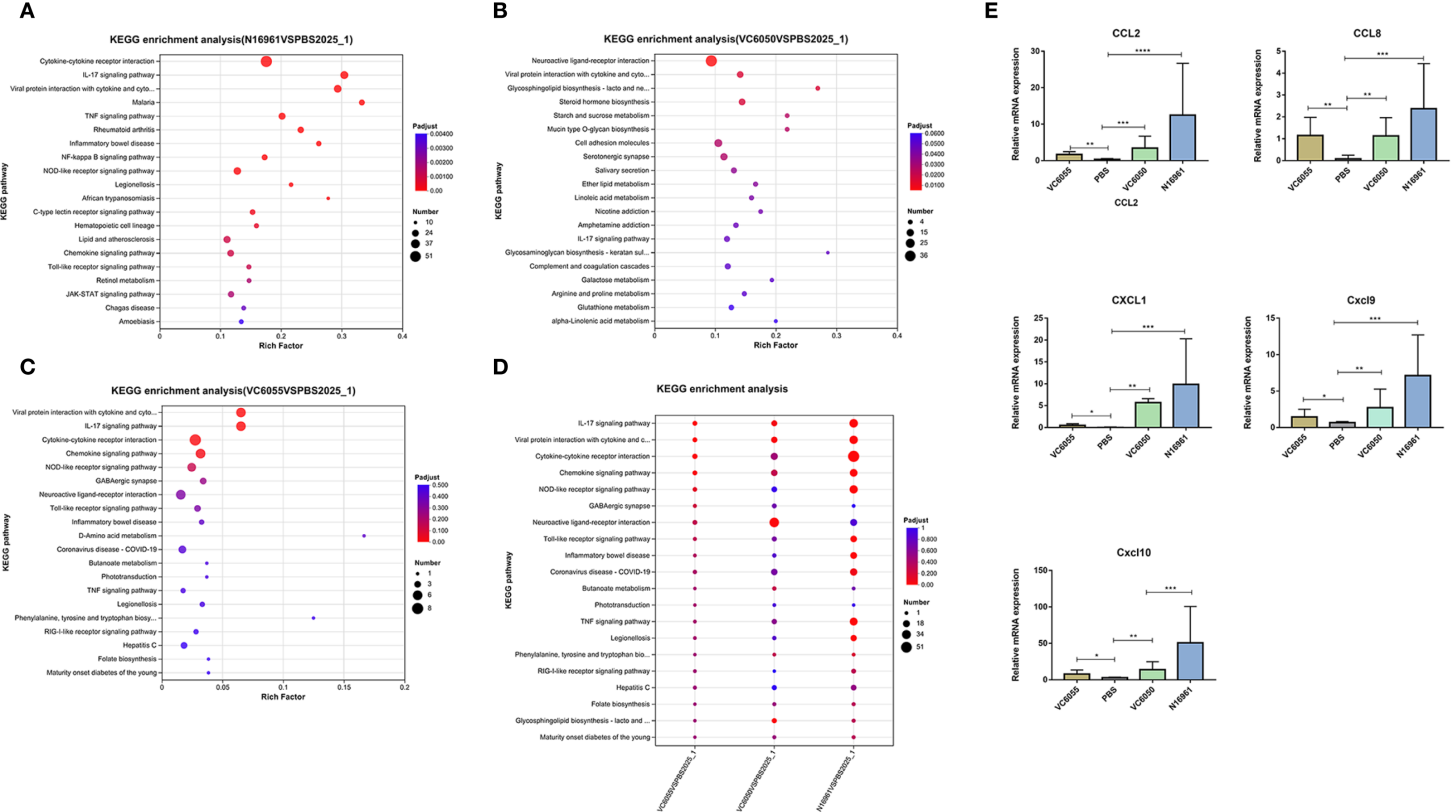
KEGG enrichment analysis results. **(A–C)** The top 20 pathways ranked by KEGG enrichment analysis for groups N16961, VC6050, and VC6055 compared to the control group (PBS), respectively. **(D)** KEGG enrichment analysis of multi-gene set for groups N16961, VC6050, and VC6055 versus PBS control. **(E)** the expression of CCL2, CCL8, CXCL1, CXCL9, and CXCL10. * P<0.01, ** P<0.001, *** P<0.0001, **** P<0.00001.

Compared to the PBS control group, the DEGs in the VC6055 group were predominantly enriched in the following pathways: viral protein interaction with cytokine and cytokine receptor, IL-17 signaling pathway, cytokine-cytokine receptor interaction, chemokine signaling pathway, NOD-like receptor signaling pathway, and GABAergic synapse (**Figure 2A**). In the VC6050 group, the DEGs were primarily enriched in neuroactive ligand-receptor interaction, viral protein interaction with cytokine and cytokine receptor, IL-17 signaling pathway, and cell adhesion molecules pathways (**Figure 2B**). For the N16961 group, the DEGs were mainly associated with cytokine-cytokine receptor interaction, IL-17 signaling pathway, viral protein interaction with cytokine and cytokine receptor, TNF signaling pathway, inflammatory bowel disease, NF-kappa B signaling pathway, and NOD-like receptor signaling pathway (**Figure 2C**). Collectively, these findings indicate that transcriptional changes in the neonatal mouse intestine were primarily associated with immune and inflammation-related genes following *V. cholerae* infection. Among these inflammation-related pathways, the expression levels of chemokines CCL2, CCL8, CXCL1, CXCL9, and CXCL10 were significantly upregulated (**Figure 2E**). Additionally, the expression of the transcriptional activator AP-1 was also markedly increased.

KEGG pathway enrichment analysis further revealed that in inflammation-related pathways, such as the IL-17 signaling pathway, cytokine-cytokine receptor interaction, and TNF signaling pathway, the number of enriched genes gradually decreased in the order of N16961, VC6050, and VC6055 groups (**Figure 2D**).

**Figure 3:**
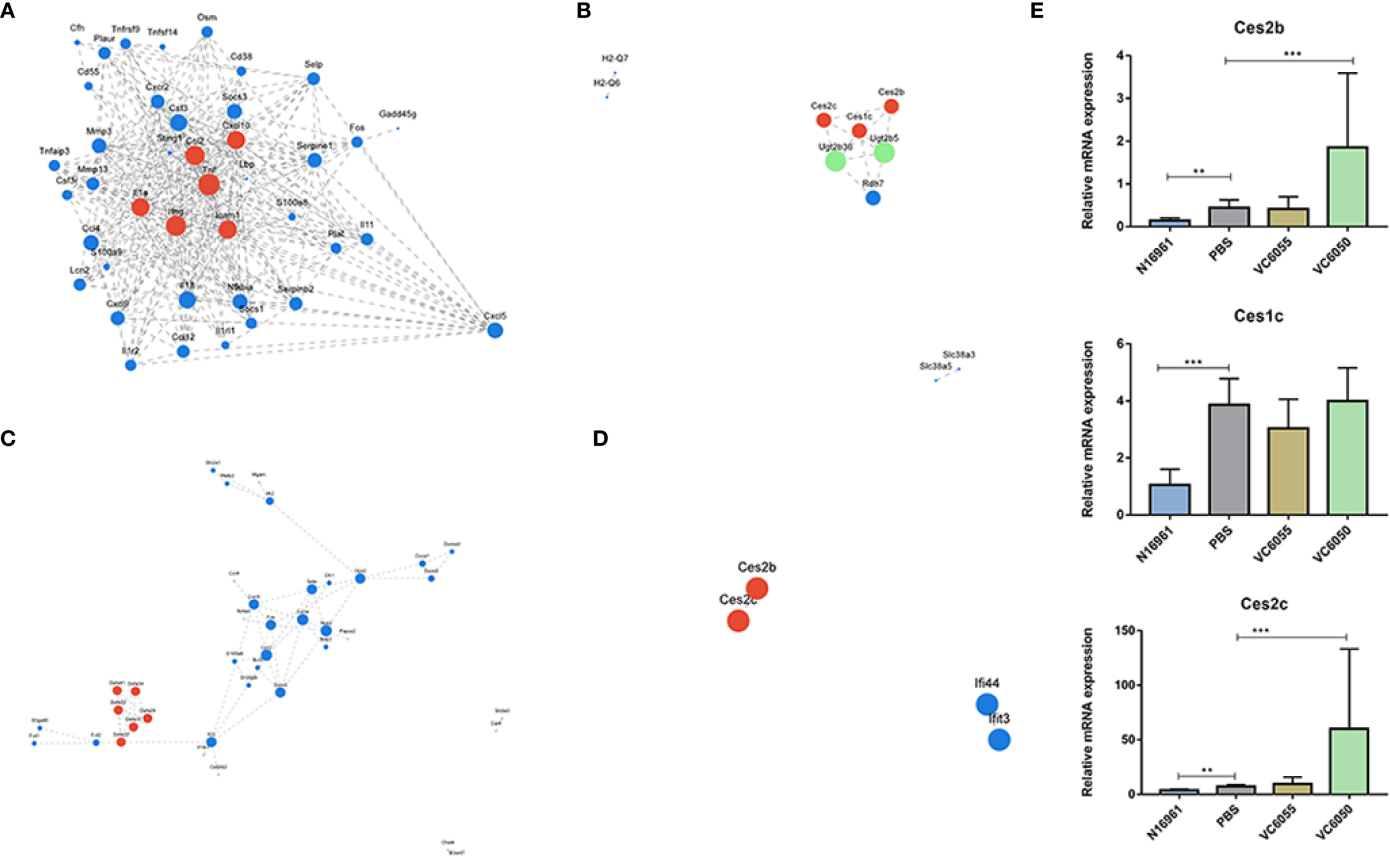
Protein protein interaction network of genes that the top 20 pathways ranked by KEGG enrichment analysis. **(A)** the up-regulated genes of N16961 versus VC6050/VC6055. **(B)** the down-regulated genes of N16961 versus VC6050/VC6055. **(C)** the up-regulated genes of N16961/VC6050 versus VC6055. **(D)** the down-regulated genes of N16961/VC6050versus VC6055. **(E)** the expression of genes encoding carboxyesterases (Ces2b, Ces1c, Ces2c). ** P<0.001, *** P<0.0001.

Compared with the N16961 infection group, the infection groups of the new lineage strains VC6050 and VC6055 shared 187 downregulated genes and 165 upregulated genes. The downregulated genes were mainly enriched in inflammation-related pathways, such as the IL-17 signaling pathway, cytokine-cytokine receptor interaction, TNF signaling pathway, and NF-kappa B signaling pathway. The PPI network analysis revealed that the main interaction network was predominantly composed of inflammation-related genes, including IL-11, IL-18, IL-1R2, TNFα, and certain chemokines, such as CXCL10, CCL2, and CCL12 (**Figure 3A**). In contrast, the PPI network constructed from the upregulated genes showed that the main interaction network included genes encoding carboxyesterases (Ces2b, Ces1c, Ces2c), genes involved in aquaporin-mediated transport (AQP8, AQP12, Gng8), and some inflammation-related genes, such as ACER1 and Cysltr1 (**Figure 3B**). Compared to the PBS control group, the expression level of carboxylesterase was decreased in the N16961 strain, increased in the VC6050 strain, and remained unchanged in the VC6055 strain (**Figure 3E**).

Compared with the infection group of the non-toxin-producing strain VC6055, the infection groups of the toxin-producing strains N16961 and VC6050 shared 250 DEGs. Among these, 177 genes were up-regulated in the toxin-producing strains, mainly enriched in pathways such as the IL-17 signaling pathway, TNF signaling pathway, NOD-like receptor signaling pathway, and transcriptional regulatory genes, including FOS, AP-1, and Sele. The PPI network analysis showed that the genes forming the primary interaction network could be classified into two major categories. The first category consisted of inflammation-related genes, such as NLRP3, CD14, IL18, IL18R1, IL22, Nupr1, Nr4a3, Cxcl5, S100a14, S100a8, and S100a9. The second category included Defa genes, such as Defa23, Defa29, Defa30, Defa34, Defa35, Defa36, Defa39 (**Figure 3C**). The down-regulated genes mainly comprised carboxyesterase genes (Ces2b, Ces2c), as well as RIKEN cDNA genes and predicted genes (**Figure 3D**).

**Figure 4:**
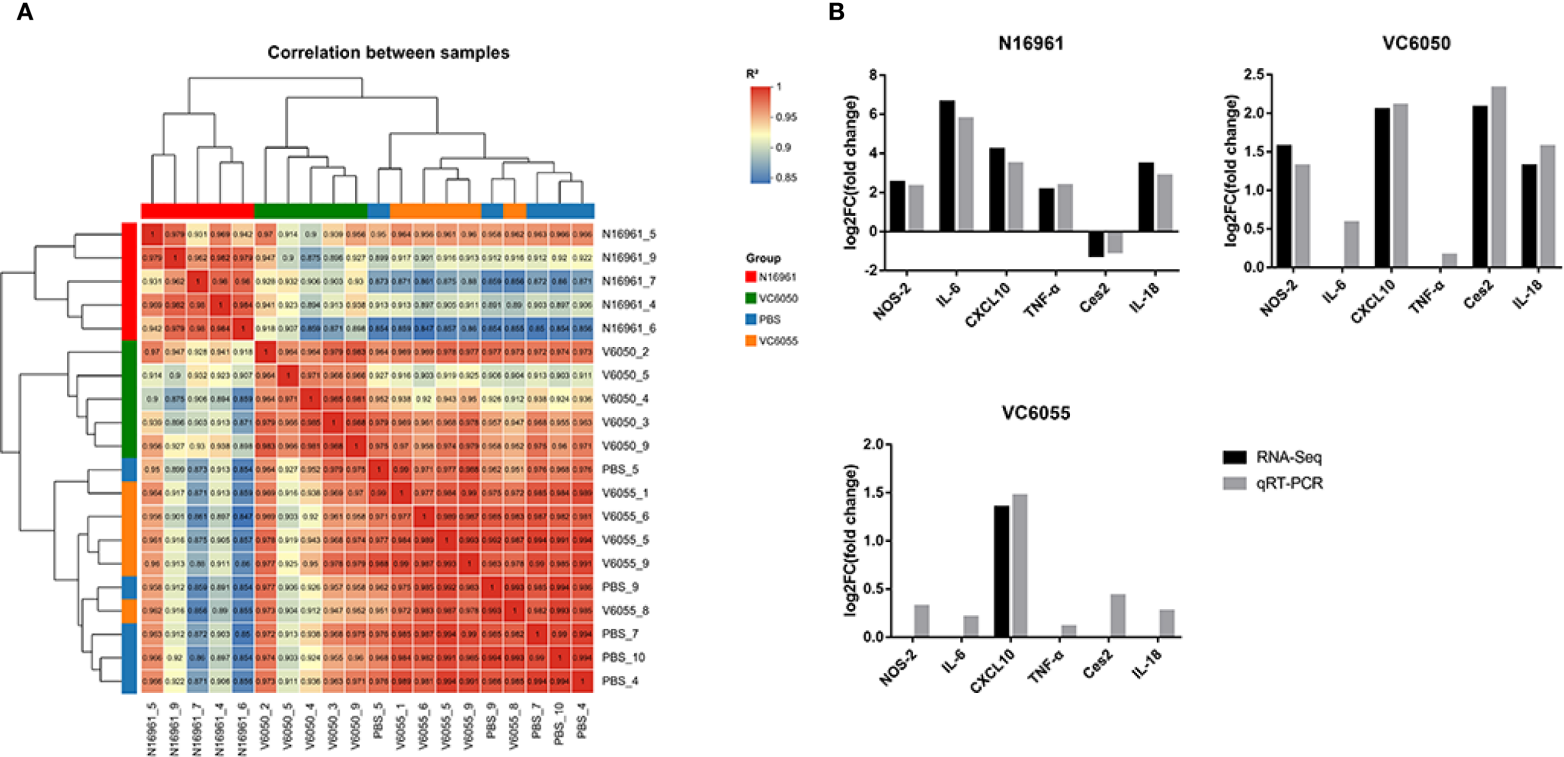
Sample correlation analysis and RT-qPCR validation of transcriptome data. **(A)** Correlation analysis of 20 samples revealed good clustering among sample groups. **(B)** The relative transcript levels of six DEGs were consistent between RNA-seq and RT-qPCR. Black bars represent RNA-seq results, while gray bars represent RT-qPCR results.

To validate the transcriptome data, we conducted a sample correlation analysis and selected six DEGs for RT-qPCR analysis. The sample correlation analysis revealed good clustering among sample groups, with only one sample deviating in the PBS and VC6055 groups (**Figure 4A**). The relative transcript levels of the six selected DEGs showed consistent trends in both RNA-seq and RT-qPCR data (**Figure 4B**).

### Cytokine levels in adult Balb/c mouse infected *V. cholerae*

**Figure 5:**
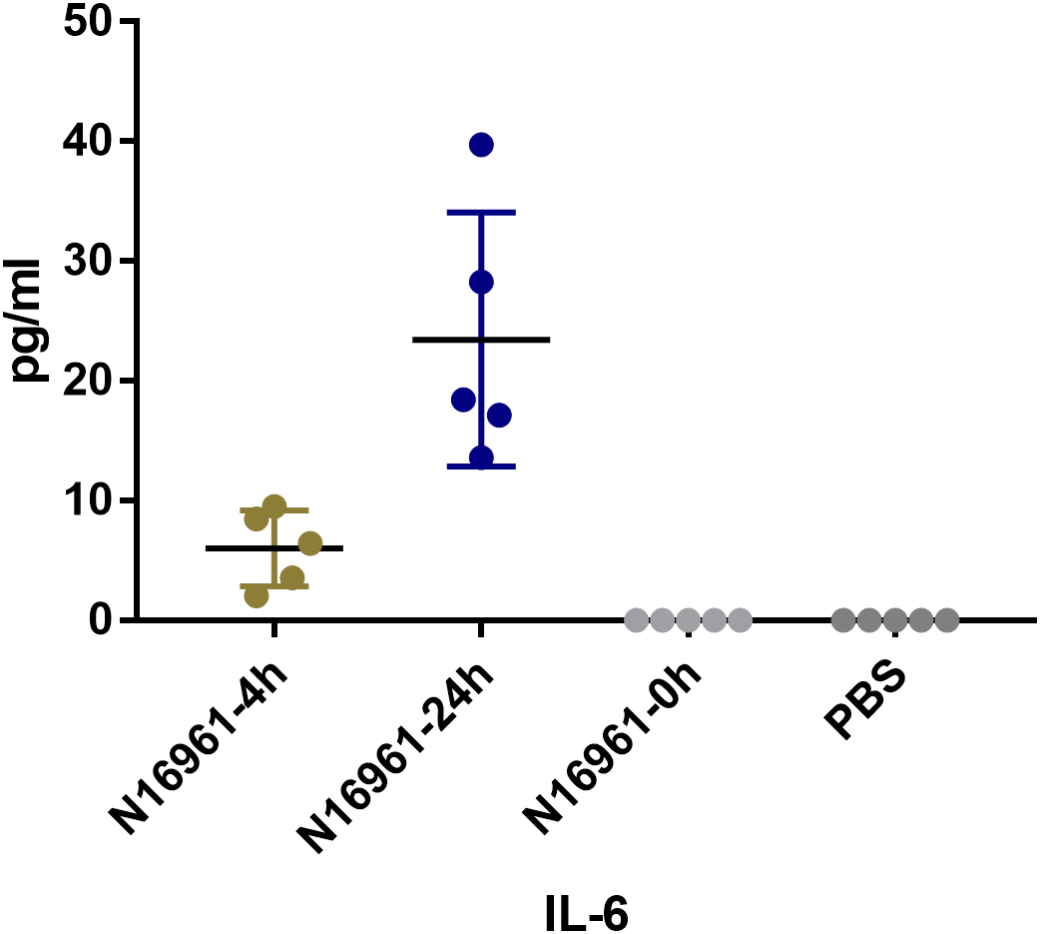
IL-6 concentration in Balb/c mice at 4h and 24h after oral inoculation with N16961. Balb/c Mice were orally infected with V. cholerae N16961. Serum samples were collected at 4 hours and 24 hours post-inoculation for cytokine measurement.

Female Balb/c mice (6–8 weeks old) were orally inoculated with *V. cholerae* strain N16961, using PBS as a control. Serum samples were collected at 4 hours and 24 hours post-inoculation for cytokine measurement. The results showed that the goodness of fit (R²) for the standard curve was above 98% (**Supplementary Figure S1**). The IL-6 concentration at 4 hours and 24 hours was significantly higher than that of the PBS control group, while the differences in other groups were not statistically significant (P > 0.05) (**Figure 5**).

## Discussion

In addition to specific immune responses, disease protection can also be mediated by the innate immune response through the heightened secretion of inflammatory mediators with bactericidal or bacteriostatic properties. In this study, adult Balb/c mice and C57BL/6 suckling mice were utilized to examine the innate immune response characteristics of the new lineage strains of *V. cholerae*. Following inoculation with *V. cholerae*, adult Balb/c mice showed no significant differences in the concentrations of cytokines IL-2, IL-4, IL-10, IL-17A, TNF, and IFN-γ, except for a significant increase in IL-6 concentration compared to the control group. This suggests that adult Balb/c mice may not be the optimal animal model for observing the immune response to *V. cholerae*, which is consistent with previous research findings (15).

To further investigate the inflammation induced by *V. cholerae*, RNA sequencing was performed on the small intestine of neonatal mice after infection. The results showed that transcript changes in the neonatal mouse intestine were primarily associated with immune-and inflammation-related genes following *V. cholerae* infection. The expression levels of chemokines CCL7, CCL17, CCL21, CXCL9, and CXCL10 were significantly upregulated. In mouse models, chemokine CCL17 is expressed by dendritic cells and is involved in the induction or enhancement of a broad spectrum of inflammatory and allergic diseases, such as atopic dermatitis and colitis (18–20). CCL17 was upregulated in specific intestinal dendritic cell (DC) subsets after infection with Salmonella (21). Similarly, CCL21 recruits dendritic cells to intestinal tissues after oral Salmonella typhimurium infection in mice (22). In the context of *V. cholerae* infection, the level of CCL21 was also increased in adult mouse pulmonary infection models (14). The upregulation of these chemokines is associated with the migration and activation of inflammatory cells in the intestine, which is consistent with the infiltration of inflammatory cells observed in intestinal tissues after *V. cholerae* infection.

To date, over thirty strains of the new lineage of serogroup O1 El Tor V. cholerae have been isolated, most of which are non-toxigenic. Genomic analysis has revealed a highly clonal population structure among these strains. Characterization of their biological properties indicated minimal variations in biofilm formation, motility, and growth (detailed findings will be published separately). The two strains selected for this study, VC6050 and VC6055, were isolated early from clinical patients and are well representative. When comparing toxin-producing strains (N16961 and VC6050) with the non-toxin-producing strain (VC6055), the expression of inflammation-related genes such as IL-22 and CXCL5 was upregulated in the toxin-producing strains. Notably, the expression of several alpha-defensin genes (Defa) was also upregulated. Alpha-defensins are important effectors of innate immunity across the plant and animal kingdoms. In the mammalian small intestine, Paneth cell alpha-defensins are antimicrobial peptides that contribute to host defense against enteric pathogens (23). Additionally, alpha-defensins serve as crucial regulators of intestinal microbial ecology (24, 25). These results indicate that compared to CTX-negative strains, CTX-positive strains may not only activate more inflammatory factors but also induce the host to produce more antimicrobial peptides.

Despite the absence of a significant difference in colonization ability between the CTX-positive strains VC6050 and N16961, the bacterial load of these strains was significantly higher than that of the CTX-negative strain VC6055 in the intestines of suckling mice. This enhanced colonization ability of CTX-positive strains may be related to the increased expression of CXCL5. CT may modulate the host response to promote colonization through the influx of neutrophils. CXCL5 (epithelial neutrophil-activating peptide-78), a pro-inflammatory chemotactic cytokine, may lead to the chemotaxis of neutrophils to the small intestine (26). In our study, multiple inflammation-related pathways were still altered in the CTX-negative strain VC6055, although the changes in inflammatory factors were weaker than those observed in the toxigenic strains N16961 and VC6050. This suggests that while the presence of CT is crucial for *V. cholerae* to cause severe diarrhea, components such as flagellin and lipopolysaccharides in CTX-negative strains can still trigger inflammatory responses and pathological changes in suckling mice (27, 28).

Compared with N16961, the expression of genes involved in aquaporin-mediated transport and carboxyesterase genes was upregulated in the new lineage strains of VC6050 and VC6055. Aquaporin (AQP) channels play a central role in regulating fluid homeostasis in the colon (29). Aquaporin 8, a member of the aquaporin family, facilitates transmembrane water transport in the mouse intestine (30). In hepatocytes, AQP8 enhances permeability through cAMP stimulation, thereby promoting water transport (31). It is speculated that the upregulation of AQP transcription may be associated with the diarrhea symptoms caused by *V. cholerae*. Carboxyesterases play an important role in drug and lipid metabolism and are widely expressed in the liver, lungs, and intestines of humans and animals (32–34). Macrophages deficient in carboxyesterase genes are more responsive to lipopolysaccharide (LPS) -induced inflammation, showing higher levels of inflammatory cytokines (35). Additionally, it has been observed that after oral administration of LPS to mice, serum carboxylesterase levels decreased by 70% (36). This is consistent with our findings that the seventh pandemic strains exhibit higher levels of inflammatory cytokines and chemokines compared to the new lineage strains of *V. cholerae*.

Overall, CTX-positive, CTX-negative, and the new lineage strains of *V. cholerae* activate specific chemokine and cytokine signaling pathways upon infecting the host, thereby influencing the host’s immune response and inflammatory reaction. The precise mechanisms of these chemokines and cytokines in *V. cholerae* infection, as well as their impact on disease severity and progression, require further investigation.

## Data Availability

The datasets presented in this study can be found in online repositories. The names of the repository/repositories and accession number(s) can be found below: https://www.ncbi.nlm.nih.gov/, PRJNA1251524.

## Supplementary material

Supplementary Figure 1 The standard curve for IL-6.

## References

[1] Kaper JB Morris JG Jr. Levine MM. Cholera. Clin Microbiol Rev. (1995) 8:48–86. doi: 10.1128/CMR.8.1.48, PMID: 7704895 PMC172849

[2] Albert MJ Siddique AK Islam MS Faruque AS Ansaruzzaman M Faruque SM. Large outbreak of clinical cholera due to Vibrio cholerae non-O1 in Bangladesh. Lancet. (1993) 341:704. doi: 10.1016/0140-6736(93)90481-u, PMID: 8095621

[3] Devault AM Golding GB Waglechner N Enk JM Kuch M Tien JH. Second-pandemic strain of Vibrio cholerae from the Philadelphia cholera outbreak of 1849. N Engl J Med. (2014) 370:334–40. doi: 10.1056/NEJMoa1308663, PMID: 24401020

[4] Didelot X Pang B Zhou Z Mccann A Ni P Li D. The role of China in the global spread of the current cholera pandemic. PloS Genet. (2015) 11:e1005072. doi: 10.1371/journal.pgen.1005072, PMID: 25768799 PMC4358972

[5] Luo Y Payne M Kaur S Octavia S Lan R. Genomic evidence of two-staged transmission of the early seventh cholera pandemic. Nat Commun. (2024) 15:8504. doi: 10.1038/s41467-024-52800-w, PMID: 39353924 PMC11445481

[6] Mutreja A Kim DW Thomson NR Connor TR Lee JH Kariuki S. Evidence for several waves of global transmission in the seventh cholera pandemic. Nature. (2011) 477:462–5. doi: 10.1038/nature10392, PMID: 21866102 PMC3736323

[7] Waldor MK Mekalanos JJ. Lysogenic conversion by a filamentous phage encoding cholera toxin. Science. (1996) 272:1910–4. doi: 10.1126/science.272.5270.1910, PMID: 8658163

[8] Gherlan GS Lazar DS Florescu SA Dirtu RM Codreanu DR Lupascu S. Non-toxigenic Vibrio cholerae - just another cause of vibriosis or a potential new pandemic? Arch Clin cases. (2025) 12:5–16. doi: 10.22551/2025.46.1201.10305, PMID: 39925986 PMC11801190

[9] Ninin E Caroff N El Kouri D Espaze E Richet H Quilici ML. Nontoxigenic vibrio Cholerae O1 bacteremia: case report and review. Eur J Clin Microbiol Infect Dis. (2000) 19:489–91. doi: 10.1007/s100960000296, PMID: 10947231

[10] Yan H Pang B Lu X Gao Z Lu P Zhang X. Cholera caused by a new clone of serogroup O1 vibrio cholerae- beijing municipality, China, June 2021. China CDC Wkly. (2022) 4:31–2. doi: 10.46234/ccdcw2021.279, PMID: 35586521 PMC8796729

[11] Qadri F Raqib R Ahmed F Rahman T Wenneras C Das SK. Increased levels of inflammatory mediators in children and adults infected with Vibrio cholerae O1 and O139. Clin Diagn Lab Immunol. (2002) 9:221–9. doi: 10.1128/cdli.9.2.221-229.2002, PMID: 11874856 PMC119937

[12] Mathan MM Chandy G Mathan VI. Ultrastructural changes in the upper small intestinal mucosa in patients with cholera. Gastroenterology. (1995) 109:422–30. doi: 10.1016/0016-5085(95)90329-1, PMID: 7615191

[13] Qadri F Wenneras C Albert MJ Hossain J Mannoor K Begum YA. Comparison of immune responses in patients infected with Vibrio cholerae O139 and O1. Infect Immun. (1997) 65:3571–6. doi: 10.1128/iai.65.9.3571-3576.1997, PMID: 9284121 PMC175508

[14] Fullner KJ Boucher JC Hanes MA Haines GK 3rd Meehan BM Walchle C. The contribution of accessory toxins of Vibrio cholerae O1 El Tor to the proinflammatory response in a murine pulmonary cholera model. J Exp Med. (2002) 195:1455–62. doi: 10.1084/jem.20020318, PMID: 12045243 PMC2193536

[15] Bishop AL Patimalla B Camilli A. Vibrio cholerae-induced inflammation in the neonatal mouse cholera model. Infect Immun. (2014) 82:2434–47. doi: 10.1128/IAI.00054-14, PMID: 24686062 PMC4019157

[16] Nygren E Li BL Holmgren J Attridge SR. Establishment of an adult mouse model for direct evaluation of the efficacy of vaccines against Vibrio cholerae. Infect Immun. (2009) 77:3475–84. doi: 10.1128/IAI.01197-08, PMID: 19470748 PMC2715679

[17] Love MI Huber W Anders S. Moderated estimation of fold change and dispersion for RNA-seq data with DESeq2. Genome Biol. (2014) 15:550. doi: 10.1186/s13059-014-0550-8, PMID: 25516281 PMC4302049

[18] Semmling V Lukacs-Kornek V Thaiss CA Quast T Hochheiser K Panzer U. Alternative cross-priming through CCL17-CCR4-mediated attraction of CTLs toward NKT cell-licensed DCs. Nat Immunol. (2010) 11:313–20. doi: 10.1038/ni.1848, PMID: 20190758

[19] Cook AD Lee MC Saleh R Khiew HW Christensen AD Achuthan A. TNF and granulocyte macrophage-colony stimulating factor interdependence mediates inflammation via CCL17. JCI Insight. (2018) 3(6):e99249. doi: 10.1172/jci.insight.99249, PMID: 29563337 PMC5926922

[20] Lee KM Jarnicki A Achuthan A Fleetwood AJ Anderson GP Ellson C. CCL17 in inflammation and pain. J Immunol. (2020) 205:213–22. doi: 10.4049/jimmunol.2000315, PMID: 32461237

[21] Erazo AB Wang N Standke L Semeniuk AD Fulle L Cengiz SC. CCL17-expressing dendritic cells in the intestine are preferentially infected by Salmonella but CCL17 plays a redundant role in systemic dissemination. Immun Inflammation Dis. (2021) 9:891–904. doi: 10.1002/iid3.445, PMID: 33945673 PMC8342217

[22] Cheminay C Schoen M Hensel M Wandersee-Steinhauser A Ritter U Korner H. Migration of Salmonella typhimurium –harboring bone marrow–derived dendritic cells towards the chemokines CCL19 and CCL21. Microb Pathog. (2002) 32:207–18. doi: 10.1006/mpat.2002.0497, PMID: 12071677

[23] Salzman NH Ghosh D Huttner KM Paterson Y Bevins CL. Protection against enteric salmonellosis in transgenic mice expressing a human intestinal defensin. Nature. (2003) 422:522–6. doi: 10.1038/nature01520, PMID: 12660734

[24] Salzman NH Hung K Haribhai D Chu H Karlsson-Sjoberg J Amir E. Enteric defensins are essential regulators of intestinal microbial ecology. Nat Immunol. (2010) 11:76–83. doi: 10.1038/ni.1825, PMID: 19855381 PMC2795796

[25] Shimizu Y Nakamura K Yoshii A Yokoi Y Kikuchi M Shinozaki R. Paneth cell alpha-defensin misfolding correlates with dysbiosis and ileitis in Crohn’s disease model mice. Life Sci Alliance. (2020) 3(6):e201900592. doi: 10.26508/lsa.201900592, PMID: 32345659 PMC7190275

[26] Koch AE Kunkel SL Harlow LA Mazarakis DD Haines GK Burdick MD. Epithelial neutrophil activating peptide-78: a novel chemotactic cytokine for neutrophils in arthritis. J Clin Invest. (1994) 94:1012–8. doi: 10.1172/JCI117414, PMID: 8083342 PMC295150

[27] Rui H Ritchie JM Bronson RT Mekalanos JJ Zhang Y Waldor MK. Reactogenicity of live-attenuated Vibrio cholerae vaccines is dependent on flagellins. Proc Natl Acad Sci U.S.A. (2010) 107:4359–64. doi: 10.1073/pnas.0915164107, PMID: 20160087 PMC2840140

[28] Shin OS Uddin T Citorik R Wang JP Della Pelle P Kradin RL. LPLUNC1 modulates innate immune responses to Vibrio cholerae. J Infect Dis. (2011) 204:1349–57. doi: 10.1093/infdis/jir544, PMID: 21900486 PMC3182310

[29] Cao Y He Y Wei C Li J Qu L Zhang H. Aquaporins alteration profiles revealed different actions of senna, sennosides, and sennoside A in diarrhea-rats. Int J Mol Sci. (2018) 19(10):E3210. doi: 10.3390/ijms19103210, PMID: 30336596 PMC6213963

[30] Dutta A Das M. Deciphering the role of aquaporins in metabolic diseases: A mini review. Am J Med Sci. (2022) 364:148–62. doi: 10.1016/j.amjms.2021.10.029, PMID: 35196511

[31] Garcia F Kierbel A Larocca MC Gradilone SA Splinter P Larusso NF. The water channel aquaporin-8 is mainly intracellular in rat hepatocytes, and its plasma membrane insertion is stimulated by cyclic AMP. J Biol Chem. (2001) 276:12147–52. doi: 10.1074/jbc.M009403200, PMID: 11278499

[32] Jones RD Taylor AM Tong EY Repa JJ. Carboxylesterases are uniquely expressed among tissues and regulated by nuclear hormone receptors in the mouse. Drug Metab Dispos. (2013) 41:40–9. doi: 10.1124/dmd.112.048397, PMID: 23011759 PMC3533427

[33] Lian J Nelson R Lehner R. Carboxylesterases in lipid metabolism: from mouse to human. Protein Cell. (2018) 9:178–95. doi: 10.1007/s13238-017-0437-z, PMID: 28677105 PMC5818367

[34] Schwer H Langmann T Daig R Becker A Aslanidis C Schmitz G. Molecular cloning and characterization of a novel putative carboxylesterase, present in human intestine and liver. Biochem Biophys Res Commun. (1997) 233:117–20. doi: 10.1006/bbrc.1997.6413, PMID: 9144407

[35] Szafran BN Borazjani A Scheaffer HL Crow JA Mcbride AM Adekanye O. Carboxylesterase 1d inactivation augments lung inflammation in mice. ACS Pharmacol Transl Sci. (2022) 5:919–31. doi: 10.1021/acsptsci.2c00098, PMID: 36268116 PMC9578131

[36] Wait R Chiesa G Parolini C Miller I Begum S Brambilla D. Reference maps of mouse serum acute-phase proteins: changes with LPS-induced inflammation and apolipoprotein A-I and A-II transgenes. Proteomics. (2005) 5:4245–53. doi: 10.1002/pmic.200401292, PMID: 16196095

