# Supplementary figures and images for: Inflammation induced by the new lineage of Vibrio cholerae serogroup O1 in the neonatal mouse model
Source: Front Immunol. 2025 Oct 3;16:1617803. doi: 10.3389/fimmu.2025.1617803 (PMC12531251; doi:10.3389/fimmu.2025.1617803)

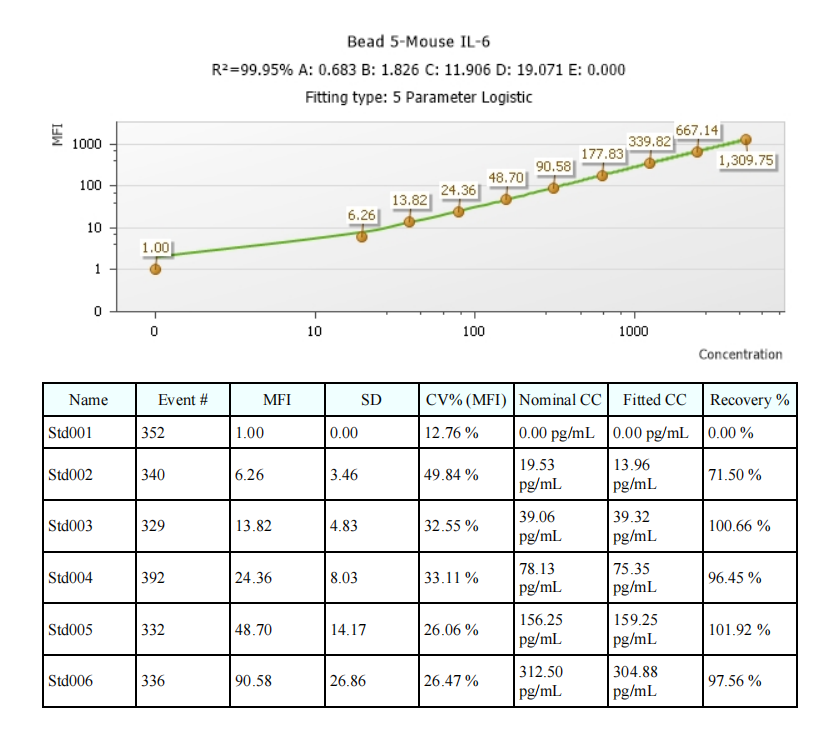

Supplement: Supplementary Figure 1 — The standard curve for IL-6. [file Image1.png]
