# Supplementary material for: Inflammation induced by the new lineage of Vibrio cholerae serogroup O1 in the neonatal mouse model
Source: Front Immunol. 2025 Oct 3;16:1617803. doi: 10.3389/fimmu.2025.1617803 (PMC12531251; doi:10.3389/fimmu.2025.1617803)
Supplement: Supplementary file 2 [file Table1.docx]

TABLE S1 primers used in the RT-qPCR reactions

| Targets | primer | Sequence (5’–3’) |
| --- | --- | --- |
| GAPDH | Forward | ACTCCCACTCTTCCACCTTC |
|  | Reverse | TCTTGCTCAGTGTCCTTGC |
| NOS-2 | Forward | ACATCGACCCGTCCACAGTAT |
|  | Reverse | CAGAGGGGTAGGCTTGTCTC |
| IL-6 | Forward | GTTCTCTGGGAAATCGTGGAAA |
|  | Reverse | AAGTGCATCATCGTTGTTCATACA |
| CXCL10 | Forward | GCCTCTCGTACATACAGACGC |
|  | Reverse | CCAGTTCTGCTTTGGATCAGC |
| TNF-α | Forward | ATGAGCACAGAAAGCATGATC |
|  | Reverse | TACAGGCTTGTCACTCGAATT |
| Ces2 | Forward | GGAGTGGTGTGAGAGATGCG |
|  | Reverse | CAGGTTAGAGCCCTCACGG |
| IL-18 | Forward | ATGCCTGATATCGACCGAAC |
|  | Reverse | TGGCACACGTTTCTGAAAGA |
